# Relationship and prognostic value of microvascular obstruction and infarct size in st-elevation myocardial infarction as visualized by magnetic resonance imaging

**DOI:** 10.1186/1532-429X-13-S1-P183

**Published:** 2011-02-02

**Authors:** Suzanne de Waha, Steffen Desch, Ingo Eitel, Georg Fuernau, Philipp Lurz, Matthias Grothoff, Matthias Gutberlet, Gerhard Schuler, Holger Thiele

**Affiliations:** 1 University of Leipzig, Heart Center, Leipzig, Germany

## Introduction

Data on the relation of infarct size and microvascular obstruction (MO) assessed with magnetic resonance imaging (MRI) as predictors for adverse clinical outcome after ST-elevation myocardial infarction (STEMI) are not consistent. In addition, the ratio of MO and infarct size might be an even stronger predictor for outcome after STEMI which has not been investigated, yet.

## Purpose

To analyze the impact of of microvascular obstruction, infarct size and the ratio of MO and infarct size as predictors for long-term clinical outcome after STEMI in a large prospective trial.

## Methods

STEMI patients reperfused by primary angioplasty (n=423) within 12 hours after symptom onset underwent contrast-enhanced-MRI at a median of 3 days after the index event (IQR 2-4). MO and infarct size were measured 15 minutes after gadolinium injection with late enhancement sequences and were analyzed quantitatively (as percentage of the left ventricular mass [%LV]). Clinical follow-up was conducted after 19 months (IQR 10-27). The primary endpoint was defined as composite of death, non-fatal myocardial reinfarction and congestive heart failure.

## Results

In comparison to MO and infarct size, the ratio of MO and infarct size was the strongest predictor for MACE in univariable analysis (hazard ratio [HR] 1.83, 95%CI 1.34-2.50, p<0.001). In a first multivariable analysis including infarct size, ejection fraction and end-systolic volume and end-diastolic volume, MO was the only parameter independently associated with MACE (HR 1.03, 95%CI 1.01-1.04, p=0.001). In a second multivariable analysis including the ratio of MO and infarct size on top of MO, infarct size, ejection fraction and end-systolic volume, and end-diastolic volume, the ratio of MO and infarct size was (HR of 2.05; 95%CI 1.01-4.14, p=0.04) identified as the strongest independent predictor for MACE.

Furthermore, inclusion of the ratio of MO and infarct size resulted in a significant increase of the C-statistics, demonstrating an incremental prognostic value above the separate analysis of both parameters (0.69 vs. 0.72, p<0.001).

## Conclusions

Although in contrast to infarct size, MO is associated with adverse clinical outcome after STEMI even after adjustment for other MRI parameters, the ratio of MO and infarct size is a more powerful predictor for long-term outcome after STEMI adding incremental prognostic value above the separate analysis of these parameters.

